# Multi-View Omnidirectional Vision and Structured Light for High-Precision Mapping and Reconstruction

**DOI:** 10.3390/s25206485

**Published:** 2025-10-20

**Authors:** Qihui Guo, Maksim A. Grigorev, Zihan Zhang, Ivan Kholodilin, Bing Li

**Affiliations:** 1 Department of Electric Drive, Mechatronics and Electromechanics, South Ural State University, Chelyabinsk 454080, Russia; asp22gt944@susu.ru (Q.G.); asp22ct750@susu.ru (Z.Z.); kholodilinii@susu.ru (I.K.); 2 Department of Automation, North China Electric Power University, Baoding 071003, China; li_bing@ncepu.edu.cn

**Keywords:** virtual omnidirectional camera model, computer vision, omnidirectional images, simulation, environment modeling, measurements

## Abstract

Omnidirectional vision systems enable panoramic perception for autonomous navigation and large-scale mapping, but physical testbeds are costly, resource-intensive, and carry operational risks. We develop a virtual simulation platform for multi-view omnidirectional vision that supports flexible camera configuration and cross-platform data streaming for efficient processing. Building on this platform, we propose and validate a reconstruction and ranging method that fuses multi-view omnidirectional images with structured-light projection. The method achieves high-precision obstacle contour reconstruction and distance estimation without extensive physical calibration or rigid hardware setups. Experiments in simulation and the real world demonstrate distance errors within 8 mm and robust performance across diverse camera configurations, highlighting the practicality of the platform for omnidirectional vision research.

## 1. Introduction

Omnidirectional vision systems provide a 360° field of view and are widely used in environmental modeling [1], submarine monitoring [2], robotic navigation [3], localization [4], and tracking [5]. Compared with conventional multi-camera systems that require image stitching, a single omnidirectional sensor captures the entire scene in one shot, improving data-collection efficiency. Fisheye cameras, in particular, are attractive for UAV imaging, indoor reconstruction, intelligent transportation, and security due to their low cost, simple structure, compact size, wide field of view, and ease of use [6,7,8,9]. Sharma et al. [10] obtained real-time omnidirectional images using coordinated multi-fisheye camera acquisition and image stitching techniques, thereby providing the high-quality environmental data required for six-degrees-of-freedom (6DoF) immersive experiences in UAV-assisted VR. In 3D indoor scene reconstruction, fisheye cameras provide comprehensive recognition of architectural elements such as floors, walls, and ceilings, thereby enabling effective reconstruction of indoor environments [11]. In intelligent transportation systems, fisheye cameras significantly enhance monitoring of complex road networks and intersections, enabling high-precision, real-time detection of vehicles and pedestrians within a 180-degree field of view [12]. Furthermore, fisheye cameras enable wide-range, blind-spot-free monitoring in home security applications through their ultra-wide-angle view, making real-time surveillance of the entire home possible [13].

Despite these advantages, fisheye lenses introduce strong radial distortion. Without proper modeling and correction [14,15], objects near the periphery are resampled with significant geometric error, degrading localization, path planning, and obstacle detection [16,17]. Robustness can be further reduced by lighting variation, noise, and cluttered backgrounds. Structured-light [18,19,20] projection alleviates these issues by encoding depth with high spatial resolution: projecting coded fringes or line patterns and observing their deformation enables submillimeter-depth recovery. However, the physical field of view of conventional structured-light systems is limited, leading to occlusions and blind spots in large or cluttered scenes. Wu et al. [21] proposed a novel method for bridge vibration analysis using laser stripe tracking, which meets the precision requirements of engineering measurements. Tan et al. [22] proposed a 3D reconstruction method that integrates a polarization camera with line-structured light for weld seam localization, effectively avoiding arc interference during line laser imaging. However, the measurement range and field of view of conventional structured-light systems are often constrained by their physical configuration. In large-scale scenes, irregular environments, or scenarios with multiple obstacles, structured-light acquisition from a single viewpoint is prone to occlusions, blind spots, and missing information [23]. To overcome this limitation, integrating structured light with multi-view omnidirectional vision has gradually emerged as a feasible solution. In recent years, researchers have explored the fusion of structured-light projection with omnidirectional vision to design compact sensors capable of achieving all-around perception and high-precision 3D measurement in large-scale environments [24]. For instance, Zhang et al. [25] proposed a panoramic monocular ranging method that incorporates structured light, thereby enhancing UAV environmental perception and distance measurement accuracy in scenarios with large maneuvers and weak textures. Similarly, Qi et al. [26] developed an indoor key-point measurement approach based on structured-light and omnidirectional vision systems, enabling high-precision measurement under wide field-of-view conditions. This method not only leverages the global coverage capability of omnidirectional vision but also utilizes multi-view structured-light data to achieve detail-level high-precision reconstruction, thereby combining wide-area coverage with high spatial resolution.

Directly deploying multi-view omnidirectional and structured-light setups in the lab is expensive and hard to reproduce. A virtual simulation platform provides controllable camera/lighting/material parameters, rapid iteration, and zero-risk testing for perception and navigation algorithms. We therefore design a Unity-based simulator with fisheye cameras, laser illumination, and robot models, plus user-friendly calibration modules and TCP/IP image streaming for cross-platform processing. Such platforms can accurately simulate the imaging process under various viewpoints, camera parameters, lighting conditions, and material properties without physical hardware, enabling algorithm verification and performance evaluation at minimal cost and zero risk. In the field of autonomous navigation, similar simulation systems have been widely used for robot perception, environment reconstruction, and path planning algorithm testing. In light of these developments, this paper presents a virtual simulation system that allows for rapid environment setup and realistic modeling of omnidirectional cameras. Using this system, we develop a multi-view omnidirectional structured-light reconstruction and ranging system for fast obstacle reconstruction and accurate localization.

## 2. Related Work

### 2.1. Virtual Simulation System

To integrate data from the structured-light reconstruction based omnidirectional vision systems, it has to be tested and debugged several times in order to meet the requirements of its application. However, in real environments, it is often difficult to accurately compute the installation parameters of cameras due to setup constraints, whereas these parameters are known and easily controlled in simulated environments. Therefore, virtual reality (VR) is emerging as a key bridge between virtual environments and the physical world. Tang et al. [27] employed UAVs to capture images of virtual environments, enabling the reconstruction of immersive and realistic virtual worlds for metaverse users. Building on this, a multi-UAV cooperative hierarchical visual perception and transmission scheme, MUL-VR, was developed to provide users with an enhanced immersive quality of experience (QoE) [28]. To enable high-quality, low-cost, and highly repeatable experimental environments, various simulators [29] have been researched and developed commercially or in the laboratory, commonly including Gazebo 9 [30], Blender 2.8 [31], UniSim R491 [32], and Unity 2022.1.9f1 [33]. Gazebo is an open-source 3D physics simulation platform developed by Open Robotics. Petrillo et al. [34] incorporated a fisheye camera into the Gazebo environment using a wide-angle sensor model to match real-world systems. However, their work focused solely on robot path planning and did not address the estimation of the camera’s intrinsic parameters. Blender is open-source 3D modeling and rendering software. Feldotto et al. [35] integrated the Robot Designer plugin within Blender, allowing researchers to simulate robotic and musculoskeletal structures. However, Blender lacks real-time interaction support, making it less suitable for dynamic experimental platforms. UniSim is a recently developed simulation platform that combines Unity’s rendering capabilities with Gazebo’s physics engine. Viecco et al. [36] used the UniSim Design platform for educational experiments such as control system design in thermal processes. However, UniSim’s closed system architecture limits customization, making it more suitable for rapid prototyping of standardized teaching and demonstration scenarios. Therefore, this work adopts Unity3D [37], a professional game engine that uses C# as its scripting language, offering both programming flexibility and realistic rendering and physics. It also allows for simulating robot obstacle detection and avoidance from the perspective of a virtual camera. Haces-Garcia et al. [38] developed an accessible robotic simulation framework using Unity and compared various data communication methods to enhance interaction capabilities. Platt et al. [39] highlighted that ROS-Unity3D supports various file types and provides a powerful scripting interface for creating custom functions, making it well-suited for complex environments. Based on the Unity3D platform, we integrated fisheye cameras, laser illumination, and industrial robots with a user-friendly interface, calibration modules, and extensible functionalities to reduce experimental risks and costs. At the same time, the virtual system can directly provide experimental ground truth such as the geometric parameters and distances of the target, thereby avoiding reliance on expensive measurement equipment and ensuring the accuracy and repeatability of the evaluation.

### 2.2. Multi-View Omnidirectional Vision Reconstruction

Multi-view reconstruction, an important research direction in computer vision, aims to fuse and restore visual information of a scene from multiple images captured at different viewpoints, thereby producing imagery with higher resolution, greater completeness, or improved geometric consistency [40]. Liu et al. [41] proposed a method for generating panoramic images from textual descriptions, evaluated on planar, 360°, and full spherical panoramas. This approach improved image quality without the need for fine-tuning. However, it relies heavily on large pre-trained models and cannot generate scene images beyond the scope of the training data. In the field of industrial robotics, Li et al. [42] employed outdoor ground robots equipped with solid-state LiDAR and gimbals to reconstruct 3D models of buildings, significantly improving omnidirectional reconstruction and ranging efficiency in complex outdoor scenarios. However, this system is mainly tailored to building environments, and its generalizability to multi-task robotic scenarios remains limited. Wu et al. [43] proposed a multi-view image reconstruction method for robotic environmental perception, achieving high-precision localization of parts in industrial robotic machining. Nevertheless, the system’s complex configuration and high cost hinder its widespread adoption. In the field of agricultural robotics, Vulpi et al. [44] proposed a multi-view RGB-D camera system to enhance the perception capability of unmanned agricultural robots, achieving omnidirectional 3D reconstruction and ranging of farmland environments. However, its performance under real-time challenges such as online navigation still requires improvement. These studies demonstrate that multi-view image registration and fusion can effectively enhance spatial coverage, reduce occlusion areas, and improve detail quality to a certain extent. Nevertheless, most of the above multi-view reconstruction approaches still rely on pinhole cameras or solid-state LiDAR and lack systematic research and optimization for omnidirectional vision, particularly for fisheye cameras. This results in notable limitations in field-of-view coverage, distortion modeling, and feature matching. Moreover, current multi-view reconstruction algorithms remain sensitive to low-texture or repetitive-texture regions, where depth prediction for featureless surfaces—such as plain walls or large metal plates—often produces extensive holes or unstable fluctuations [45]. They also perform poorly in scenarios with near-zero parallax; when parallax approaches zero—such as when the camera is nearly perpendicular to a planar surface or when the baseline is very small—multi-view stereo matching cannot provide sufficient geometric constraints [46].

Against this background, we propose an obstacle contour reconstruction algorithm that integrates multi-view omnidirectional cameras with structured light. The proposed method does not require sample pre-training or explicit feature matching; instead, it fuses information directly from images captured by multi-view cameras to simultaneously achieve shape reconstruction and distance estimation, thereby reducing computational overhead. Compared with conventional multi-view visual reconstruction systems, our approach does not impose strict constraints on camera installation positions. Instead, the parameters of each omnidirectional camera can be flexibly configured as needed, while maintaining computational accuracy even in low-texture and small-parallax scenarios.

## 3. Virtual Simulation System

**Figure 1:**
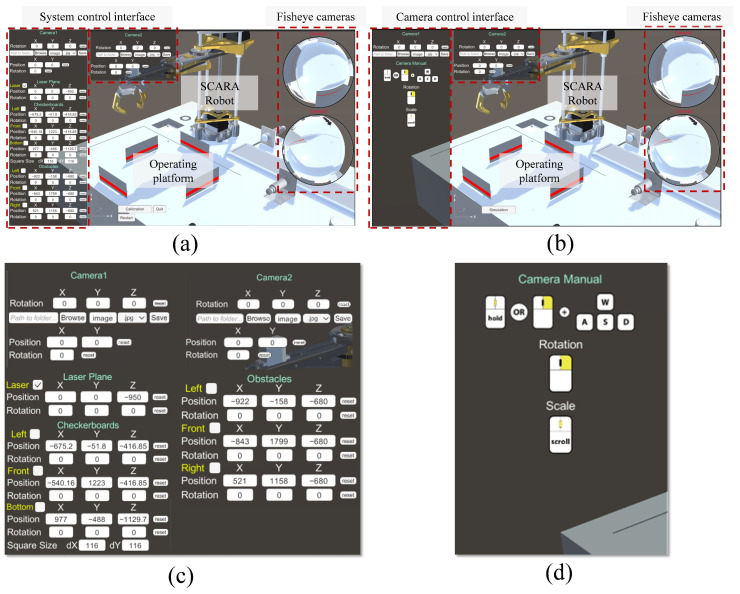
Two modules of the simulation system. (**a**) Experimental module. (**b**) Camera adjustment module. (**c**) Zoomed sections of (**a**). (**d**) Zoomed sections of (**b**).

In real-world settings, building an omnidirectional vision testbed requires substantial investment in equipment, installation, and training. By contrast, a virtual environment eliminates expensive hardware and dedicated floor space, while mitigating safety risks. Our simulation system aims to provide an economical, reliable platform for testing omnidirectional vision concepts and accelerating their validation. Compared with traditional physical platforms, our virtual simulation system offers a simpler setup process, faster response time, and lower hardware requirements for the host computer. The multi-view omnidirectional vision simulation system presented in this paper is built on the Unity3D platform and operates on Windows, supporting diverse testing scenarios across different simulated environments. The components of the virtual platform are illustrated in Figure 1. Figure 1a depicts the experimental module, where users can conveniently and efficiently modify the parameters of instruments such as the fisheye camera, laser plane, calibration board, and obstacles via a custom-designed user interface. The right side of the interface displays real-time images captured by the fisheye camera. Figure 1b shows the dynamic adjustment module for the virtual fisheye camera, where users can interactively modify camera parameters such as resolution and field of view (FOV) using mouse controls. In this study, the FOV of the virtual fisheye camera is set to 180°.

**Figure 2:**
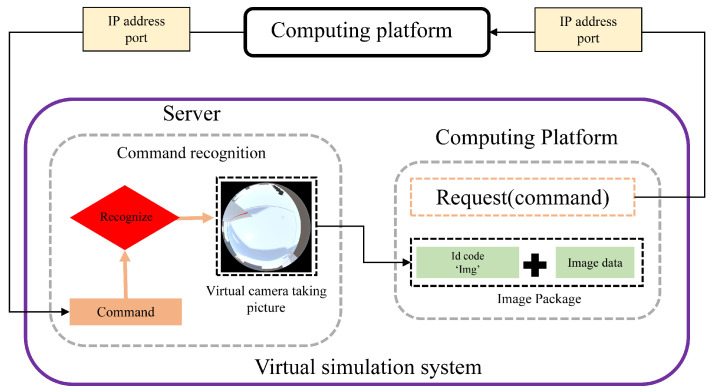
Principleof omnidirectional visual image cross-platform transmission.

Furthermore, to facilitate real-time cross-platform computational processing of images acquired by the virtual camera, we implemented a TCP-based data reception server and transmission client within the simulation system. The architecture of this setup is illustrated in Figure 2. Under this communication architecture, when the simulation system receives image acquisition commands from external platforms, the images captured by the virtual camera are packaged and loaded into the client module within the simulation system. The client establishes a network connection using the TCP/IP protocol and transmits the data to a target server on the designated external platform. The only required input parameters for this transmission method are the IP address and port number of the destination device, allowing users to easily invoke and customize communication as needed.

## 4. Reconstruction Method

The structured-light ranging method based on the omnidirectional vision system captures the structured light projected onto the surface of an obstacle and rapidly computes the distance between the obstacle and the system’s central point using a structured-light plane projection transformation equation. The corresponding projection transformation equation is given as follows:

(1) $$\left[\begin{array}{c} u\\ v\\ f(\rho ) \end{array}\right]\times \left[\begin{array}{ccc} r_{1}^{c} & r_{2}^{c} & r_{3}^{c} \end{array}\right]\left[\begin{array}{cccc} r_{1}^{l} & r_{2}^{l} & r_{3}^{l} & t^{l} \end{array}\right]\left[\begin{array}{c} X\\ Y\\ Z\\ 1 \end{array}\right]=0$$

where *u* and *v* represent the pixel coordinates of the image point; $r_{1}^{c}$, $r_{2}^{c}$, and $r_{3}^{c}$ represent the camera rotation matrix and are 3×1 column vectors; $r_{1}^{l}$, $r_{2}^{l}$, $r_{3}^{l}$, and $t^{l}$ represent the laser plane transformation matrix and are 3×1 column vectors; and *X*, *Y*, and *Z* indicate the world coordinates of the laser projection. × denotes the vector cross product.

In Equation (1), f(ρ) can be expressed as

(2) $$f\left(\rho \right)=a_{0}+a_{2}\rho^{2}+\cdots +a_{n}\rho^{n}$$

(3) $$\rho =\sqrt{{\left(u-u_{c}\right)}^{2}+{\left(v-v_{c}\right)}^{2}}$$

where $a_{n}$ is the coefficients, *N* is the degree of the polynomial, and $u_{c}$ and $v_{c}$ represent the coordinates’ center of an omnidirectional image.

Since the distance between the laser plane and the optical center of the camera is constant and known, the Z-axis value of the reconstructed data in the omnidirectional vision system remains unchanged. Therefore, in Equation (1), Z=0, and the equation can be simplified as follows:

(4) $$\left[\begin{array}{c} u\\ v\\ f(\rho ) \end{array}\right]\times \left[\begin{array}{ccc} r_{1}^{c} & r_{2}^{c} & r_{3}^{c} \end{array}\right]\left[\begin{array}{ccc} r_{1}^{l} & r_{2}^{l} & t^{l} \end{array}\right]\left[\begin{array}{c} X\\ Y\\ 1 \end{array}\right]=0$$

In Equation (4), the first and second elements of the vector $t^{l}$ represent the distances between the structured-light plane and the origin of the camera coordinate system. In the 2D projection of the structured light, the laser plane remains fixed and does not change during the reconstruction process. Therefore, when the distances to the structured-light plane and the camera position parameters are known, the equation can be further simplified as follows:

(5) $$\left[\begin{array}{c} u\\ v\\ f(\rho ) \end{array}\right]\times \left[\begin{array}{ccc} h_{1}^{i} & h_{2}^{i} & h_{3}^{i} \end{array}\right]\left[\begin{array}{c} X\\ Y\\ 1 \end{array}\right]=0$$

By simplifying Equation (5), it can be expressed as

(6) $$\left[\begin{array}{c} u\\ v\\ f(\rho ) \end{array}\right]\times \left[\begin{array}{ccc} h_{1}^{i} & h_{2}^{i} & h_{3}^{i} \end{array}\right]\left[\begin{array}{c} X\\ Y\\ 1 \end{array}\right]=\left[\begin{array}{c} u\\ v\\ f(\rho ) \end{array}\right]\times \left[\begin{array}{c} h_{11}X+h_{12}Y+h_{13}\\ h_{21}X+h_{22}Y+h_{23}\\ h_{31}X+h_{32}Y+h_{33} \end{array}\right]=0$$

The result of the vector cross product can then be expressed as follows:

(7) $$v\left(h_{31}X+h_{32}Y+h_{33}\right)-f\left(\rho \right)\left(h_{21}X+h_{22}Y+h_{23}\right)=0$$

(8) $$f\left(\rho \right)\left(h_{11}X+h_{12}Y+h_{13}\right)-u\left(h_{31}X+h_{32}Y+h_{33}\right)=0$$

(9) $$u\left(h_{21}X+h_{22}Y+h_{23}\right)-v\left(h_{11}X+h_{12}Y+h_{13}\right)=0$$

Finally, by combining the above equations, the real-world coordinates reconstructed from the structured light in the camera coordinate system can be obtained as

(10) $$\left\{\begin{array}{c} X=\left(-c_{1}-b_{1}Y\right)/a_{1}\;\\ Y=\left(a_{2}c_{1}-a_{1}c_{2}\right)/\left(a_{1}b_{2}-a_{2}b_{1}\right) \end{array}\right.$$

where $a_{1}=f\left(\rho \right)h_{11}-uh_{31}$, $b_{1}=f\left(\rho \right)h_{12}-uh_{32}$, $c_{1}=f\left(\rho \right)h_{13}-uh_{33}$, $a_{2}=uh_{21}-vh_{11}$, $b_{2}=uh_{22}-vh_{12}$, and $c_{2}=uh_{23}-vh_{13}$.

**Figure 3:**
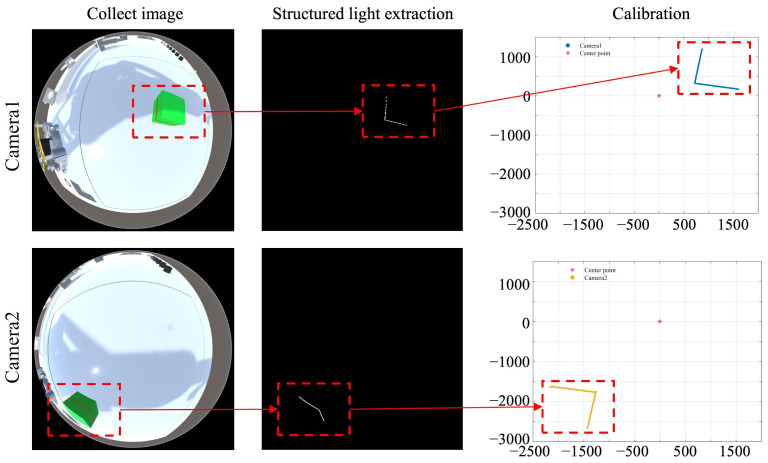
Obstacle ranging results with different omnidirectional camera viewing angles.

The transformation effect of the omnidirectional vision system using the above equations is illustrated in Figure 3. This method effectively eliminates distortions introduced by the omnidirectional imaging process and enables accurate reconstruction of the structured light. However, due to the absence of coordinate system transformation, the reconstructed results from different camera systems vary under different viewing angles, even though the actual coordinates of the observed object remain unchanged. This discrepancy prevents the integration of data collected from multiple cameras.

In multi-camera systems, to unify the reconstructed data, we define a global coordinate origin based on the principles of robot coordinate system construction. This origin is independent of both vision systems and also serves as the base frame of the robot. Within this coordinate system, it is sufficient to determine the position and orientation (rotation angles) of cameras to integrate their independently reconstructed data into a unified system. The rotation angle between the world coordinate system and the camera coordinate system is defined as θ; thus, the structured-light coordinate matrix after rotation transformation is given by

(11) $$M=R_{cv}\cdot \left[X;Y;1\right]$$

$R_{cv}$ denotes the rotation matrix of the camera coordinate system relative to the world coordinate system, and X and Y are the structured-light coordinates obtained in the camera coordinate system, as derived in Equation (10). The rotation matrix $R_{cv}$ is defined as

(12) $$R_{cv}=\left[\begin{array}{ccc} \cos(\theta ) & -\sin(\theta ) & 0\\ \sin(\theta ) & \cos(\theta ) & 0\\ 0 & 0 & 1 \end{array}\right]$$

After the rotational transformation, a final correction is required based on the position of the camera’s optical center in the world coordinate system. The corrected coordinates are computed as follows:

(13) $$\left[\begin{array}{c} x\\ y \end{array}\right]=M+\left[\begin{array}{c} x_{cv}\\ y_{cv} \end{array}\right]$$

where $x_{cv}$ and $y_{cv}$ represent the coordinates of the fisheye camera within the unified coordinate system, while x and y denote the transformed structured-light coordinates in the same system.

**Figure 4:**
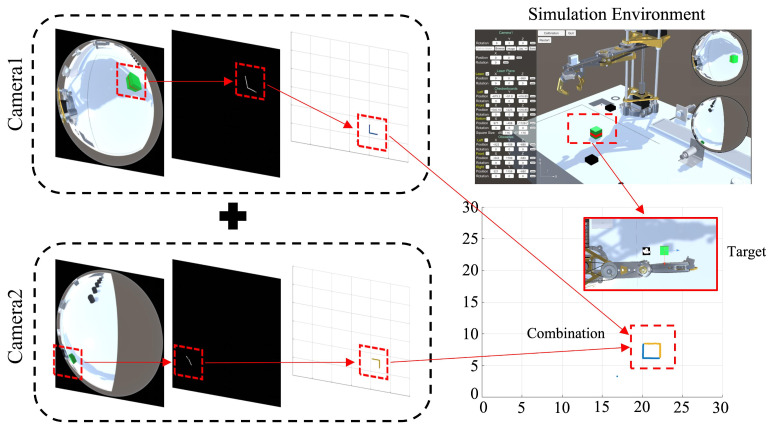
Data integration process of the multi-view omnidirectional vision system. The **left** part illustrates the omnidirectional vision data reconstruction process; the **right** part shows the multi-view data fusion results.

Through the above transformation process, the independently reconstructed structured-light data from the vision system are mapped into a unified coordinate system. The overall transformation workflow is illustrated in Figure 4. By overlaying the transformed reconstruction results, a more complete representation of the obstacle can be obtained. Building upon the existing ranging capability, data collected from multiple cameras can more accurately reconstruct the external structure of the target object and expand the perception of surrounding environmental geometry.

## 5. Experimental Results and Analysis

To evaluate the performance of the developed virtual simulation system and the proposed multi-view omnidirectional vision-based data reconstruction method, a series of tests were conducted from multiple perspectives, including target reconstruction accuracy, obstacle ranging precision, and effectiveness in real-world scenarios. The traditional monocular omnidirectional vision system [47] and the DUSt3R method [48] for target 3D reconstruction based on multiple images were used as the control group for comparative analysis. Since the DUSt3R method is designed for conventional RGB camera images, we incorporated regular cameras into the virtual system with position parameters identical to those of the fisheye cameras, ensuring consistent viewing angles. The captured conventional images were then used as input for DUSt3R. The virtual simulation system was run on the Windows platform, using an AMD Ryzen 5 5600U CPU @ 2.30 GHz (Advanced Micro Devices, Santa Clara, CA, USA) with 6 cores and 16 GB of RAM. In the real-world experiments, the test platform consisted of a 650 nm line laser generator and an HF890 industrial fisheye camera.

### 5.1. Target Reconstruction Performance

Compared with conventional planar vision systems, omnidirectional vision offers a wider field of view, enabling the capture of more comprehensive environmental information. However, when capturing target objects, a single omnidirectional camera often fails to obtain complete data due to occlusions or limited viewing angles. This is especially evident in structured-light capture of polygonal objects, where partial data loss leads to incomplete shape reconstruction. In contrast, the multi-view omnidirectional vision system allows for multi-angle capture of the target, resulting in more complete structural imaging. To validate the superiority of our proposed omnidirectional vision reconstruction method, two sets of object reconstruction experiments were conducted. The first set of experiments focused on reconstructing the same object at different distances, while the second set examined the reconstruction of objects with varying shapes.

#### 5.1.1. Target Reconstruction Performance at Different Distances

**Figure 5:**
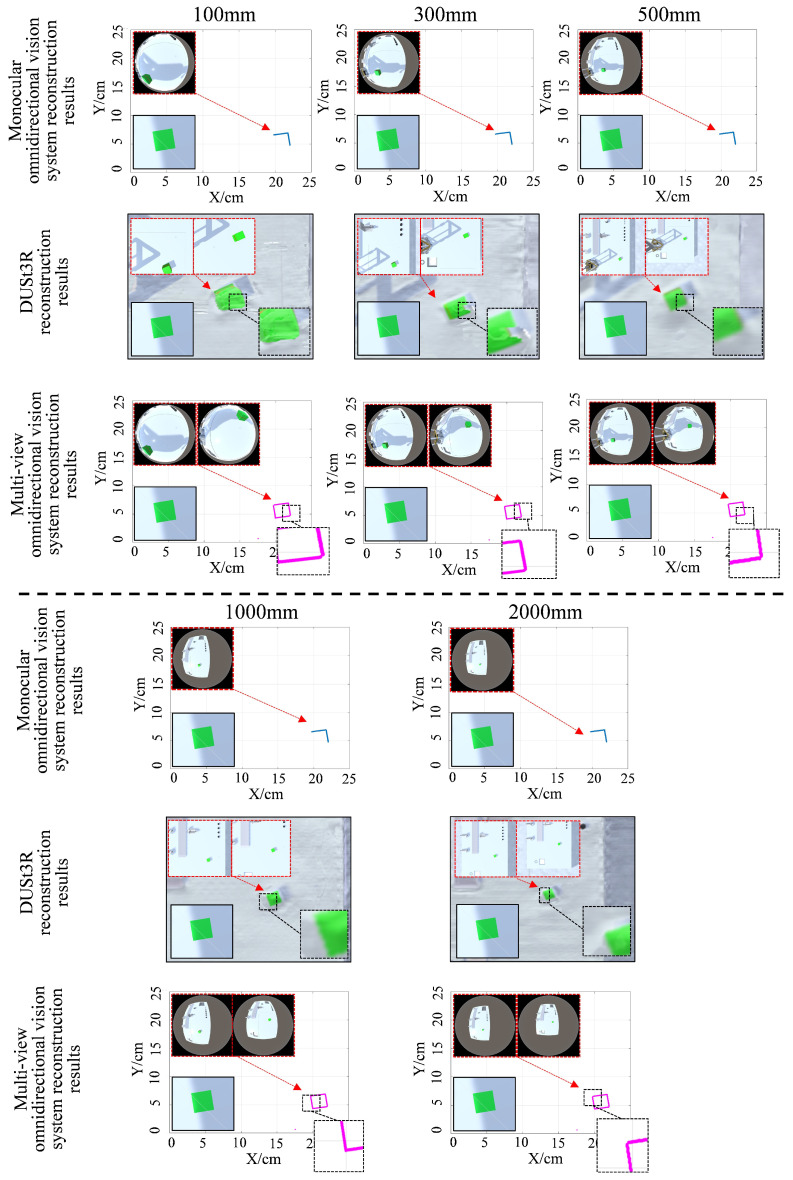
Reconstruction results of the target object at different distances with different methods. For the monocular omnidirectional vision and multi-view omnidirectional vision methods, the input images were captured by fisheye cameras; for DUSt3R, the input images were captured by conventional perspective cameras.

To evaluate the reconstruction performance of the proposed method at varying distances, the omnidirectional vision system was positioned at three heights above the laser plane: 100 mm, 300 mm, and 600 mm. The test object was a cube with a side length of 20 mm. Experimental results obtained using different methods are shown in Figure 5. As illustrated, the dual-view omnidirectional vision system outperforms both the monocular system and DUSt3R, achieving a more complete reconstruction of the target obstacle’s shape. DUSt3R possesses strong global modeling capabilities, allowing it to establish pixel-wise correspondences across the entire image. This enables the reconstruction of all objects within the scene. However, when only two input images are provided, its reconstruction accuracy for smaller objects is limited. At a height of 300 mm, the reconstructed target cube exhibited a noticeable gap.

To quantitatively assess the shape reconstruction accuracy of both methods, we adopted two evaluation metrics: Average Symmetric Surface Distance (ASSD) [49] and Intersection over Union (IoU) [50]. ASSD measures the average minimum pixel-wise distance between points on two contour sets, reflecting their geometric similarity. A lower ASSD value indicates better reconstruction accuracy. The calculation is defined as follows:

(14) $$ASSD=\frac{1}{|A|+|B|}\left(\sum_{a\in A}d\left(a,B\right)+\sum_{b\in B}d\left(A,b\right)\right)$$

where *A* denotes the set of contour points from the ground-truth object, and *B* represents the set of reconstructed contour points. d(A,b) refers to the minimum distance from point b∈B to set *A*, and d(a,B) is the minimum distance from point a∈A to set *B*, computed as

(15) $$d\left(a,B\right)=\min_{b\in B}∥a-b∥$$

The IoU quantifies the overlap between the reconstructed region and the ground-truth region, calculated as the ratio of their intersection area to their union area. Its value ranges from 0 to 1, with values closer to 1 indicating higher reconstruction accuracy. The equation is defined as

(16) $$IoU=\frac{Area(A\cap B)}{Area(A\cup B)}$$

**Figure 6:**
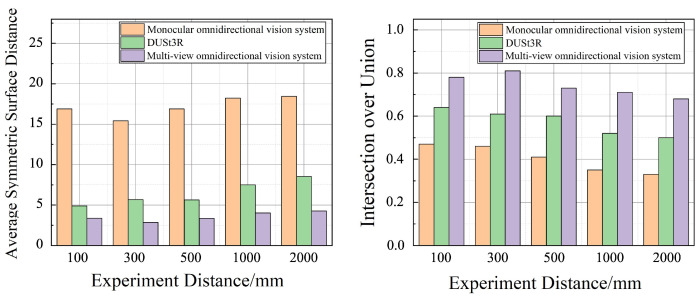
Comparison of reconstruction accuracy at different distances using different methods.

Based on the above equations, the reconstruction accuracy of object shapes for different methods in the tests presented in Figure 5 is quantitatively compared in Figure 6. In reconstruction tests at varying distances, measurement error generally increases as the distance between the camera and the target object increases, resulting in a gradual decline in reconstruction accuracy. However, the multi-view omnidirectional vision system consistently demonstrates significantly higher reconstruction accuracy compared to the monocular system and DUSt3R method. Moreover, it exhibits greater stability in reconstruction performance across different camera heights. Even under long-distance conditions (e.g., 1000 mm and 2000 mm), where DUSt3R shows a marked decrease in reconstruction accuracy due to reduced image detail, the proposed system maintains relatively robust performance by leveraging the wide field of view of the fisheye camera to reliably capture laser information.

#### 5.1.2. Reconstruction Performance for Objects with Different Shapes

**Figure 7:**
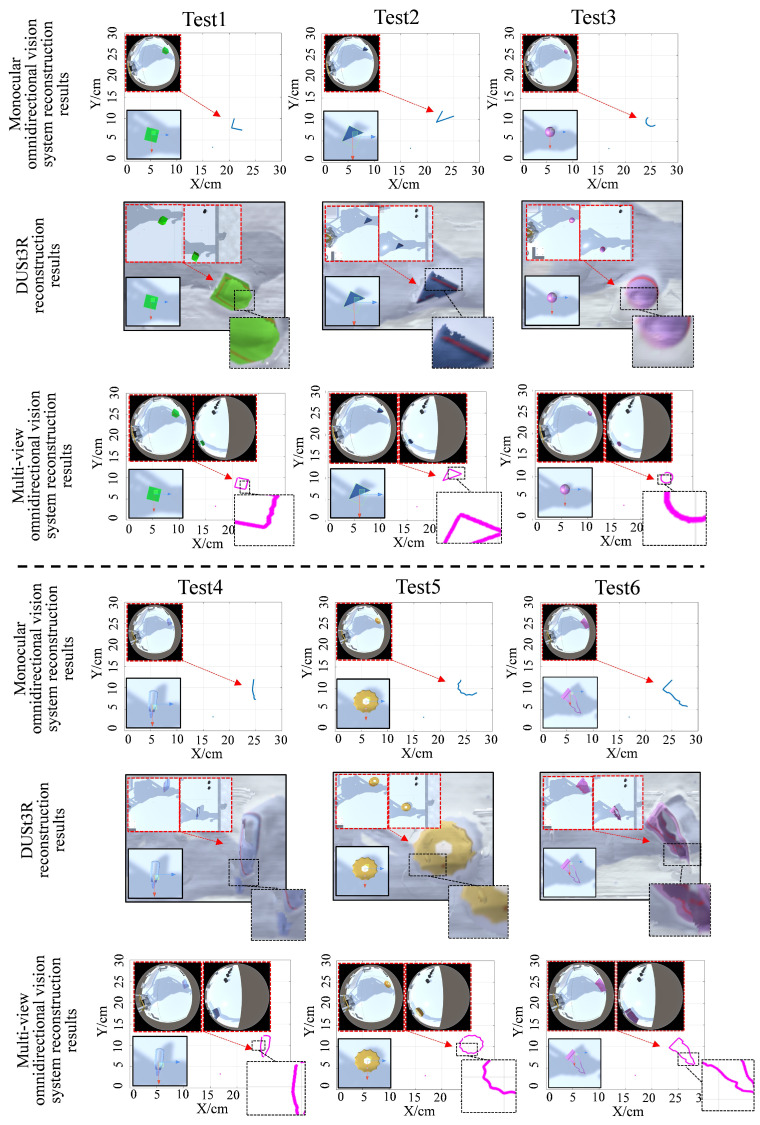
Reconstruction results of objects with different shapes at the same height using different methods. For the monocular omnidirectional vision and multi-view omnidirectional vision methods, the input images were captured by fisheye cameras; for DUSt3R, the input images were captured by conventional perspective cameras.

To evaluate the system’s capability in reconstructing objects of various shapes, six models were selected in the virtual testing environment, including three regular-shaped models and three complex curved-surface models. The objects were reconstructed using both the monocular vision systems, the DUSt3R and multi-view omnidirectional vision systems, and the reconstruction results were compared. The experimental results are shown in Figure 7. It can be observed that the monocular omnidirectional system captured limited structured-light data, resulting in only partial reconstruction of the target objects. When dealing with regular geometric shapes, DUSt3R maintains good reconstruction performance, with reconstructed contours closely matching the target object. However, its performance degrades when reconstructing objects with complex curved surfaces. In Test 4, the bottom part of the reconstructed tweezer model was obscured by the background; in Test 5, the gear model exhibited blurred reconstruction in the toothed regions. In contrast, the multi-view omnidirectional system successfully captured structured-light data for both regular and irregular objects, integrated the data, and achieved complete shape reconstruction. Even when reconstructing objects with complex shapes, no significant data loss was observed in the reconstruction results.

**Figure 8:**
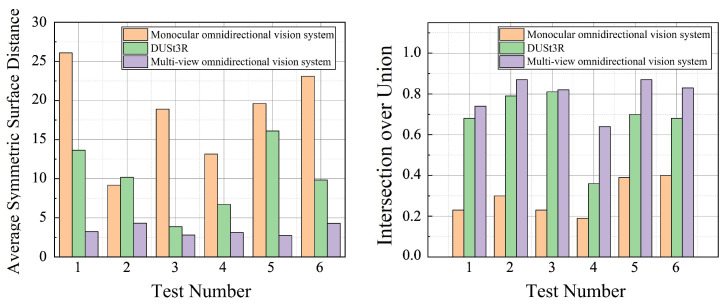
Accuracy comparison of reconstruction results for objects of different shapes using the different methods.

Figure 8 presents a comparison of reconstruction accuracy between different methods across all six test cases. In each case, the multi-view system outperforms the monocular system and DUSt3R. When reconstructing objects of various shapes, the monocular system yielded an average ASSD of 18.34, and the average ASSD of DUSt3R was 10.06, while the multi-view system achieved a significantly lower average ASSD of 3.42, indicating better overall alignment accuracy and fewer reconstruction errors. Similarly, in terms of the IoU metric, the monocular system achieved an average value of 0.29. The average value of DUSt3R was 0.67, with a noticeable decrease in performance when reconstructing objects with complex shapes, whereas the multi-view system reached an average of 0.80, demonstrating better global contour matching and reduced boundary deviation in the reconstructed results. Furthermore, when handling reconstruction of geometrically diverse and complex shapes, the multi-view system exhibited superior stability, with consistently high reconstruction accuracy across different object forms.

Table 1 presents the runtime comparison of different algorithms over six experiments on objects of varying shapes. As DUSt3R performs global space reconstruction, it imposes higher demands on computing hardware; on our experimental platform, its average runtime was 6.883 s, which was significantly higher than that of the monocular omnidirectional vision method and the proposed method. Although the proposed multi-view omnidirectional reconstruction method required an additional 0.112 s on average compared with the monocular omnidirectional vision method, it achieved a significant improvement in reconstruction performance.

### 5.2. Distance Measurement Accuracy Experiments

One of the key functions of the combined omnidirectional vision and structured-light system is obstacle distance measurement. To compare the measurement accuracy of different vision systems, six groups of distance measurement experiments were conducted. In each experiment, obstacles were placed in the left, right, forward, and back directions relative to the fisheye camera to assess the system’s measurement accuracy in various orientations. The ground-truth distances from the obstacles to the origin of the unified coordinate system are listed in Table 2. Additionally, the positions of the fisheye cameras and standard cameras were randomly altered in each experimental group, with the varying parameters summarized in Table 3, to evaluate the robustness of each vision system under different conditions.

**Figure 9:**
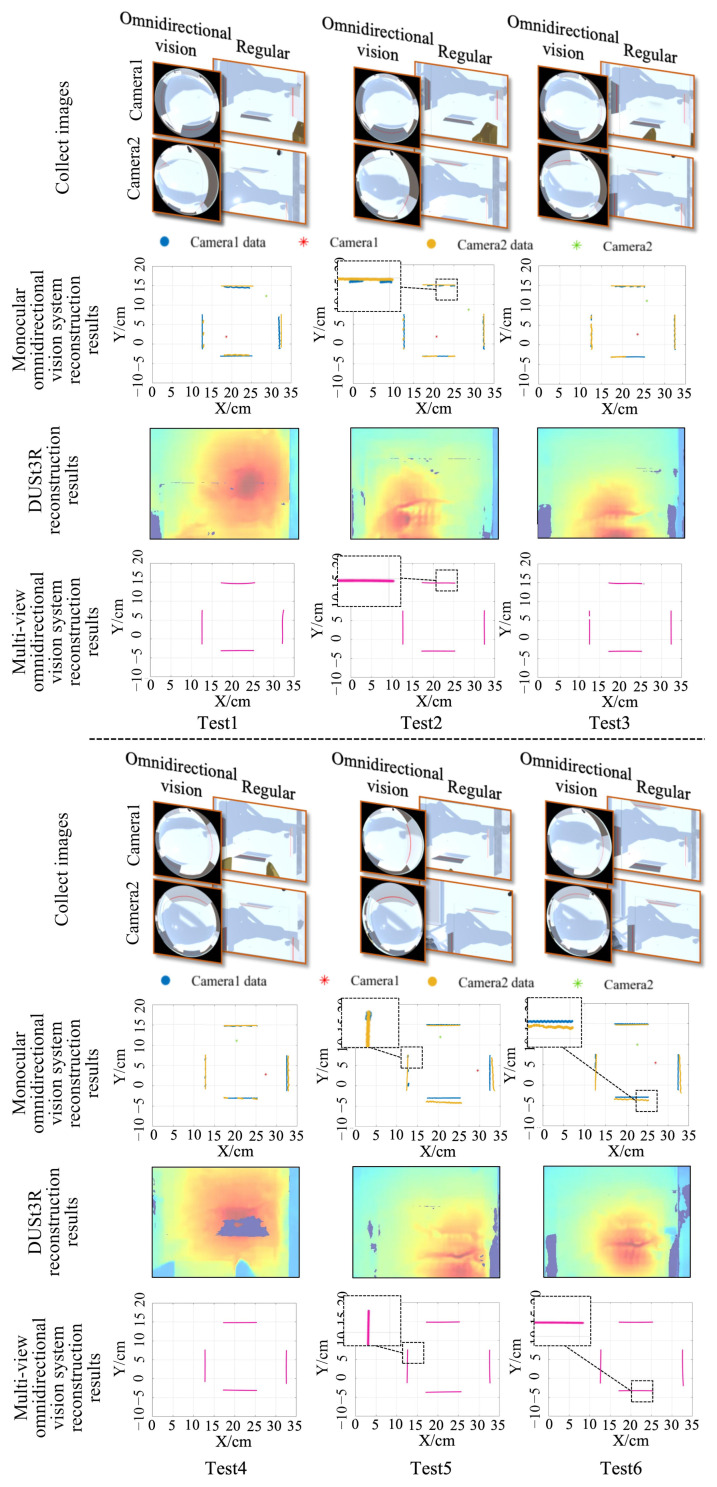
Distance measurement results of the different methods under varying camera positions and rotation angles with a fixed height. In the row of collected images, the omnidirectional vision images serve as the input for both the monocular and multi-view omnidirectional vision methods; the regular perspective images are used as the input for DUSt3R.

Figure 9 shows the experimental results for the three methods. As the position of the cameras changed, the monocular omnidirectional system was significantly affected by environmental variations, often resulting in partial loss of structured-light data. Moreover, data collected by Camera 1 and Camera 2 in the four directions (left, right, forward and back) showed noticeable discrepancies across different experiments, particularly in Test 5 and Test 6, where significant differences were observed in the right and back directions. DUSt3R is essentially based on texture matching and self-supervised modeling between images to estimate depth. In the experiments, the obstacle board featured a large, low-texture, and smooth surface, lacking sufficient texture points for reliable matching. This resulted in high uncertainty during depth estimation by the Transformer model, leading to sparse or failed correspondence matching. Consequently, DUSt3R failed to accurately recover the depth information of the test board. These results suggest that for target distance measurement tasks, both data completeness and measurement accuracy are highly sensitive to environmental conditions. Therefore, the optimal placement and orientation of cameras must be carefully considered to ensure reliable measurements. In contrast, the multi-view omnidirectional vision system integrates data from two independent fisheye cameras, enabling more complete scene reconstruction. Moreover, this method does not rely on pixel-wise feature matching between images, allowing it to accurately estimate distance information even when the target lacks surface texture features. No structural data loss was observed across six experiments, demonstrating that the proposed method exhibits greater adaptability to changes in camera positioning.

In the previous experiments, we investigated how variations in camera position and rotation affect the reconstruction accuracy of multi-view omnidirectional images. However, the distance between the fisheye camera and the structured-light plane, and the camera height, remained constant. To examine the effect of vertical installation height on ranging accuracy, we fixed the planar positions of both cameras and varied only their installation heights. The camera parameter settings were held constant throughout the tests, as detailed in Table 4. The vertical distances between the fisheye cameras and the structured-light plane used in the experiments are listed in Table 5.

**Figure 10:**
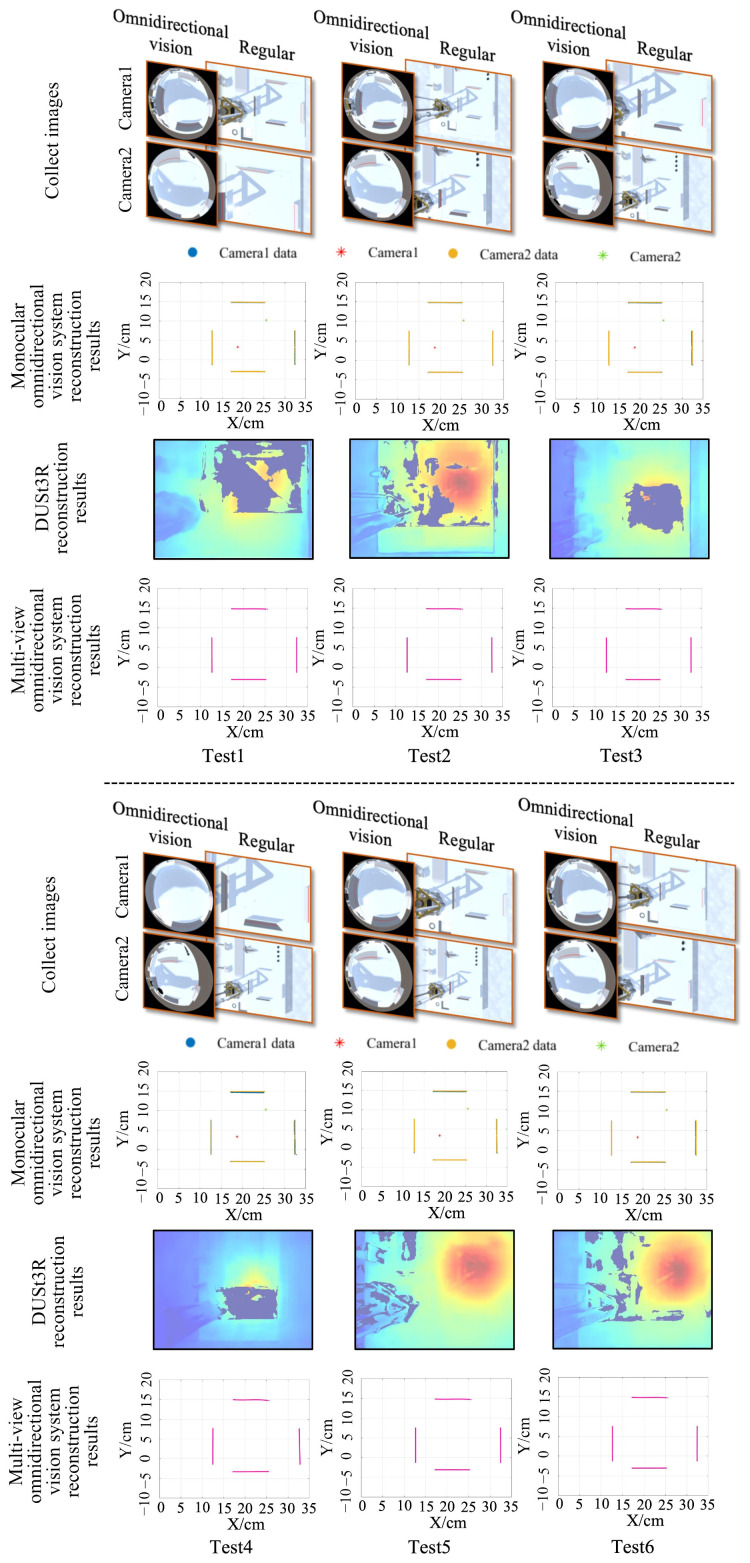
Distance measurement results of different methods at different camera installation heights with fixed positions and angles. In the row of collected images, the omnidirectional vision images serve as the input for both the monocular and multi-view omnidirectional vision methods; the regular perspective images are used as the input for DUSt3R.

Figure 10 shows the comparison results for the six test groups. Both omnidirectional vision systems successfully reconstructed the structural information of the real obstacles without the data loss previously observed in the monocular system. Although the fixed rotation angles of the two omnidirectional cameras improved DUSt3R’s depth estimation capability, it still failed to recover the depth of the test board. Both omnidirectional vision systems also demonstrated good alignment in the reconstructed data, with no significant structural deviations.

**Figure 11:**
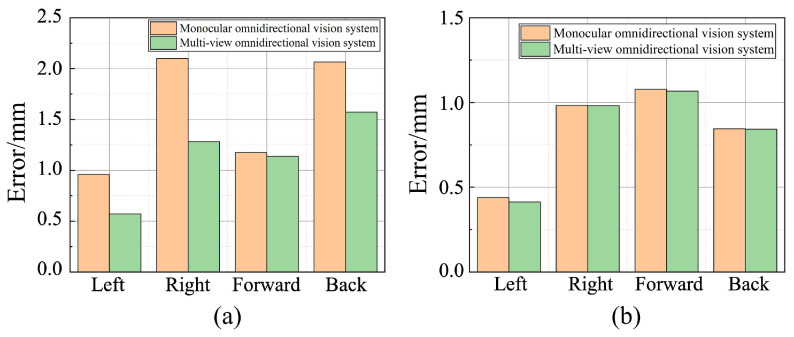
Comparison of ranging errors under different camera installation conditions. (**a**) Random camera position and orientation. (**b**) Random camera height.

Figure 11a presents the comparison of average distance measurements across six tests using different methods under varying camera positions and rotation angles with a fixed height. In the row of collected images Since DUSt3R failed to estimate the depth of the test board, the comparison was conducted only between the monocular and multi-view omnidirectional vision systems. In all four directions, the multi-view omnidirectional system achieved significantly lower measurement errors. Due to the integration of information from fisheye cameras at different viewpoints, its measurement errors were reduced by 40.4%, 38.9%, 3.1%, and 23.9% in the left, right, forward, and back directions, respectively. Even with randomly changing camera positions, the overall measurement error remained below 1.6 mm, demonstrating the superior robustness of the multi-view omnidirectional vision system. Figure 11b presents the average ranging results in four directions for both omnidirectional vision systems at different camera installation heights with fixed positions and angles. The measurement errors of the two systems were similar in all directions. However, the multi-view omnidirectional system achieved error reductions of 5.88%, 0.08%, 0.93%, and 0.39% in the left, right, front, and rear directions, respectively, compared to the monocular system. These results suggest that the vertical distance between the fisheye camera and the structured-light plane has minimal impact on the reconstruction and ranging accuracy of the multi-view omnidirectional system.

### 5.3. Real-World Experimental Results

In the previous sections, we compared the reconstruction and ranging performance of different methods within a virtual environment. To assess their practical applicability, a real-world multi-view omnidirectional vision system was constructed based on the configuration used in the virtual simulation platform. A red laser emitter was used as the structured-light source, and the target object was a cube with 1 cm edge length. By randomly placing the cube at different locations on the test platform, the multi-view omnidirectional systems were used to perform 2D reconstruction and coordinate estimation, where the estimated cube position was computed as the average of the reconstructed contour point coordinates. Six experiments were conducted. Due to DUSt3R’s suboptimal performance in close-range depth estimation, only the monocular omnidirectional vision system was used as the baseline for comparison. The installation parameters of the fisheye cameras used in the real-world experiments are listed in Table 6, obtained through the calibration method proposed by I. Kholodilin et al. [47].

**Figure 12:**
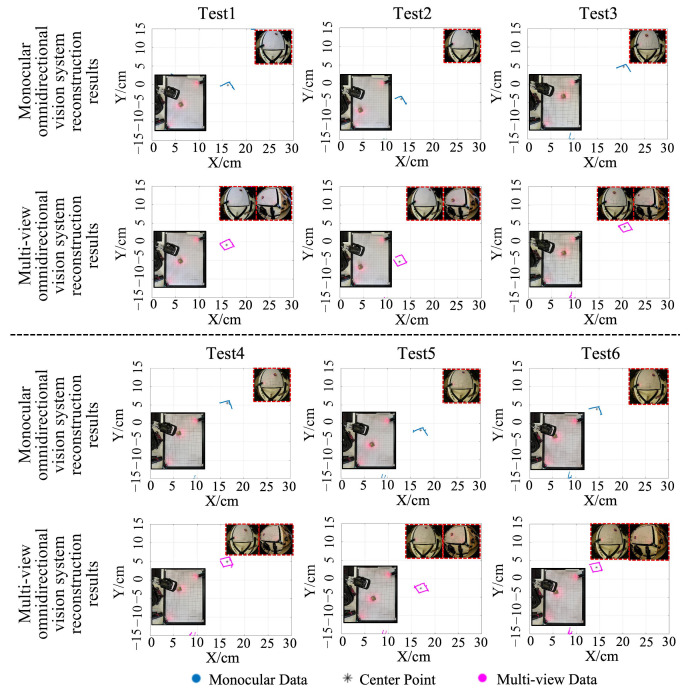
Reconstruction results of obstacles at different positions using omnidirectional vision systems in real-world experiments.

Table 7 provides the ground-truth positions of the cube and the corresponding localization results from both systems. Figure 12 shows the reconstruction results for the cube using the two systems. As observed in the virtual experiments, the monocular omnidirectional system produced incomplete reconstruction due to its limited viewing angle. In this experiment, the view of Camera 1 was limited to the upper portion of the cube, causing the monocular system to significantly overestimate the y coordinate of the object’s position. In contrast, the multi-view omnidirectional system produced x and y coordinate estimates without significant deviation from the ground truth.

**Figure 13:**
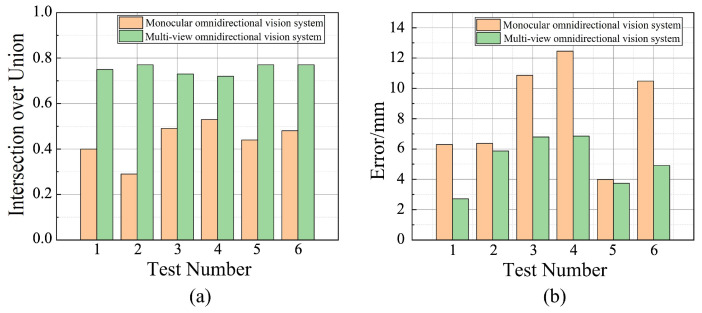
Comparison of experimental results for omnidirectional vision systems in real-world testing. (**a**) Reconstruction accuracy comparison. (**b**) Localization accuracy comparison.

Figure 13a presents the reconstruction accuracy results for both systems in the real-world tests. When reconstructing the target object at different positions, the multi-view system achieved a significantly higher average IoU of 0.752, closely matching the results obtained in the simulation experiments. Figure 13b shows the localization error comparison of the target points for both systems across all experiment groups. In all six experiments, the target point errors computed by the multi-view system were consistently smaller than those from the monocular system. On average, the measurement error was reduced by 38.93% compared to the monocular omnidirectional system. These results demonstrate that in real-world applications, the developed multi-view omnidirectional vision system can effectively enhance reconstruction and localization accuracy, with performance closely aligned to that observed in simulation tests.

Table 8 presents the runtime comparison of the two methods across six test groups. It can be observed that, compared with the virtual environment, the computation time in real-world experiments increases slightly due to additional environmental disturbances. However, the average runtime of both methods remains around 1 s, indicating that the proposed method can still maintain good reconstruction accuracy in real-world conditions with only a small computational overhead.

## 6. Conclusions

This paper presents a virtual simulation system designed for testing and evaluating omnidirectional vision systems. Based on this simulation platform, a structured-light-based data reconstruction method is proposed, enabling high-precision obstacle shape reconstruction and distance localization using multi-view omnidirectional images. The simulation system provides a controllable and accurate testing environment, allowing safe experimentation even in the absence of physical hardware resources. The proposed reconstruction and ranging method features a simplified hardware configuration, requiring only a laser emitter in addition to the omnidirectional cameras. Moreover, the system eliminates the need for complex and rigid camera installation procedures. Each camera can be flexibly configured according to the application scenario, including parameters such as mounting position and orientation. To validate the accuracy and robustness of the proposed reconstruction method, extensive experiments and analyses were conducted. The experimental results demonstrate that the proposed multi-view omnidirectional reconstruction system significantly outperforms the monocular system in both reconstruction fidelity and ranging accuracy. Furthermore, the system exhibits strong robustness to varying camera installation configurations. In real-world validation experiments, the developed multi-view omnidirectional system successfully reconstructed target objects at various positions and accurately computed their center coordinates, thereby verifying the practical effectiveness of the proposed algorithm.

Although the proposed multi-view omnidirectional reconstruction method demonstrates high accuracy and robustness in both virtual simulation and real-world environments, it has so far been validated mainly in relatively small-scale experimental scenarios and with static objects. Further research and validation are required for larger and more complex environments, especially those involving dynamic obstacles. Future work will focus on extending the system to large-scale dynamic environments and exploring its potential applications in moving obstacle detection and real-time path planning. In addition, future research will further compare the proposed algorithm with the latest structured-light and omnidirectional vision systems, conducting quantitative evaluations not only in terms of computational efficiency and reconstruction accuracy, but also assessing adaptability and robustness in complex scenarios, thereby providing a more comprehensive validation of the effectiveness and applicability of the proposed method.

## Figures and Tables

**Table 1:** Running time comparison of different methods for object shape reconstruction across six test groups in virtual experiments.

	Test 1/s	Test 2/s	Test 3/s	Test 4/s	Test 5/s	Test 6/s	Average/s
Monocular omnidirectional vision system	0.5519	0.6149	0.6147	0.9268	0.6817	0.9063	0.7161
DUSt3R	6.8	7.5	6.6	6.8	6.7	6.9	6.8833
Multi-view omnidirectional vision system	0.6666	0.6399	0.6971	0.9487	1.0550	0.9613	0.8281

**Table 2:** Obstacle parameter configuration.

Parameters	Left /mm	Right /mm	Forward /mm	Back /mm	Camera-Laser Distance/mm
Value	126.25	325.28	149.1	−31.45	95

**Table 3:** Fisheye camera parameter configuration.

Test Number	System	Camera Information				
		x/mm	y/mm	Pitch/Degrees	Roll/Degrees	Yaw/Degrees
1	camera 1	186.56	18.61	−3	1	5
	camera 2	287.08	123.10	1	−2	2
2	camera 1	207.11	18.61	2	−1	0
	camera 2	287.08	86.65	2	0	−2
3	camera 1	235.70	26.39	1	−1	−3
	camera 2	258.04	110.95	2	0	−2
4	camera 1	295.34	37.32	1	−4	2
	camera 2	205.10	119.70	3	6	5
5	camera 1	270.33	54.57	0	−4	2
	camera 2	226.77	98.80	3	4	6

**Table 4:** Camera parameters for each system.

System	Camera Information				
	x/mm	y/mm	Pitch/Degrees	Roll/Degrees	Yaw/Degrees
Camera 1	187.68	32.95	0	0	0
Camera 2	255.81	102.45	0	0	0

**Table 5:** Installation heights of fisheye cameras.

Test Number	1	2	3	4	5	6
Distance of Camera 1/mm	195	255	116	68	145	195
Distance of Camera 2/mm	95	145	235	285	205	165

**Table 6:** Real-world fisheye camera installation parameters.

System	Camera Information					Camera–Laser Distance/mm
	x/mm	y/mm	Pitch/Degrees	Roll/Degrees	Yaw/Degrees	
Camera 1	140	194	1	3	1	131
Camera 2	−122	100	4	2	2	131

**Table 7:** Real-world positioning results.

Test Number	1		2		3		4		5		6	
	x /mm	y /mm	x /mm	y /mm	x /mm	y /mm	x /mm	y /mm	x /mm	y /mm	x /mm	y /mm
Real	161	−8	129	−47	208	36	167	43	172	−23	147	28
Single	161.2	−1.71	131.15	−41	205.71	46.62	164.91	55.28	171.44	−19.06	145.27	38.34
Multi-view	158.3	−8.25	126.52	−52.32	204.25	41.66	163.57	48.93	169.76	−25.99	143.71	31.64

**Table 8:** Running time comparison of different methods for object shape reconstruction across six test groups in real-world experiments.

	Test 1/s	Test 2/s	Test 3/s	Test 4/s	Test 5/s	Test 6/s	Average/s
Monocular omnidirectional vision system	0.8315	0.9752	0.9547	0.9162	1.2774	0.8762	0.9719
Multi-view omnidirectional vision system	0.9916	1.0280	0.9861	0.9665	1.5773	0.9156	1.0775

## Data Availability

The original contributions presented in the study are included in the article; further inquiries can be directed to the corresponding author.
